# Attenuation of Endoplasmic Reticulum Stress Enhances Carvacrol-Induced Apoptosis in Osteosarcoma Cell Lines

**DOI:** 10.3390/life13030744

**Published:** 2023-03-09

**Authors:** Kuan-Wei Chiu, Hsuan-Ying Chen, Chiu-Liang Chen, Cheng-Pu Hsieh, Yi-Fu Huang

**Affiliations:** 1 Department of Orthopedic Surgery, Changhua Christian Hospital, Changhua 500209, Taiwan; 2 Orthopedics & Sports Medicine Laboratory, Changhua Christian Hospital, Changhua 500010, Taiwan; 3 Department of Nursing, Hung Kuang University, Taichung 433304, Taiwan; 4 Department of Post-Baccalaureate Medicine, College of Medicine, National Chung Hsing University, Taichung 402202, Taiwan; 5 Department of Kinesiology, Health and Leisure Studies, Chienkuo Technology University, Changhua 500020, Taiwan

**Keywords:** apoptosis, carvacrol, ER stress, osteosarcoma

## Abstract

Carvacrol is a monoterpenoid phenol that has excellent antimicrobial, antiviral, and anti-inflammatory activities. It can also improve wound healing. However, few studies have explored its antitumor effect on osteosarcoma. In this report, we tried to determine the potential efficacy of carvacrol against osteosarcoma cell lines. Our data revealed that carvacrol exposure inhibited the proliferation of osteosarcoma HOS and U-2 OS cells. In addition, carvacrol exposure enhanced the levels of cleaved PARP and caspase 3 and increased annexin V-positive cells, indicating that carvacrol exposure triggers apoptosis in osteosarcoma cell lines. Furthermore, the levels of reactive oxygen species (ROS) were enhanced after carvacrol exposure and cotreatment with NAC, the ROS scavenger, decreased the levels of cleaved PARP and caspase 3, suggesting the involvement of ROS in carvacrol-induced apoptosis. Importantly, we found that carvacrol exposure triggered several protein expressions related to endoplasmic reticulum (ER) stress, including GRP78/Bip, IRE1a, PERK, and CHOP, in HOS and U-2 OS cells, indicating that carvacrol exposure could result in ER stress in these cell lines. Cotreatment with the ER stress inhibitor 4-PBA increased the levels of cleaved PARP and caspase 3 and further suppressed cellular proliferation in carvacrol-exposed osteosarcoma cell lines. Overall, the results indicate that induced ER stress can protect cells from apoptosis, but increased ROS contributes to apoptosis in carvacrol-treated cells. In this report, we first demonstrate the role of ER stress in carvacrol-induced apoptosis and suggest that ER stress could be targeted to enhance the antitumor activity of carvacrol in osteosarcoma cell lines.

## 1. Introduction

Osteosarcoma is the most common bone cancer and predominantly occurs in adolescents and children. It is characterized by the presence of malignant mesenchymal cells producing osteoid or immature bone. Most patients suffer from pain and swelling or lumps in the involved region. Osteosarcoma may form a persistent course with local and systemic disease progression and result in death if not treated. The estimated incidence rate of osteosarcoma is eight cases/million/year in children younger than 20 years of age [1,2]. The risk factors of osteosarcoma include young age, greater height, radiation treatment for other types of cancers, and Paget disease of the bone. In addition, certain inherited cancer syndromes are associated with increased risk of developing osteosarcoma, including hereditary retinoblastoma, Li–Fraumeni syndrome, Rothmund–Thomson syndrome, Bloom syndrome, Werner syndrome, and Diamond–Blackfan anemia. Some gene mutations, such as p53 and RB, that have been identified in these diseases are further suggested to be involved in development of osteosarcoma [2,3].

Although osteosarcoma could occur in any bone, about 75% of osteosarcoma occurs near the metaphysis of long extremity bones. The most common locations include the distal femur, proximal tibia, and proximal humerus. Osteosarcoma also occurs at the axial skeleton, such as the pelvis (around 8%), chest region (around 2%), and vertebral column (around 3%), and occurs at other sites, including mandible (around 4%) and skull (around 5%) [2]. A recent study showed that nonextremity osteosarcoma and proximal osteosarcoma are correlated with poor prognosis in osteosarcoma patients [4].

Surgical resection is a major treatment for osteosarcoma. However, surgery alone is not sufficient for patients with osteosarcomas. The survival rate for those who receive surgery alone is only about 15%. In the mid-1970s, multidrug chemotherapy was introduced as adjuvant therapy to facilitate surgical removal. The common chemotherapy drugs for osteosarcoma include high-dose methotrexate, doxorubicin, cisplatin, ifosfamide, gemcitabine, decitabine, etoposide, and vincristine. This incorporation has resulted in overall five-year survival rates of 50 to 60% for patients with osteosarcoma [5,6]. Unfortunately, this outcome is nearly unchanged over the last decade. In addition, for patients who suffer from disease relapse, the outcome is very poor, with approximately 20 to 30% or less chance of survival [7,8]. Moreover, in several reported studies, chemotherapy that was used for treating the first relapse was not associated with survival after recurrence [7,9,10]. Therefore, the development of effective drugs for patients suffering from osteosarcoma is needed.

Plant-based natural products are a rich source of new therapeutic drugs and may exhibit less adverse effects compared to synthetic chemicals. Recent literature has demonstrated the anticancer activity of plant extracts or their isolates against different cancer cells, such as osteosarcoma. Hederoside C is a pentacyclic triterpene saponin, which can be isolated from Pulsatilla koreana Nakai, a traditional medicinal plant in China and Korea. Park et al. showed that hederoside C suppressed cell proliferation in human osteosarcoma cell lines by modulating MAPKs and STAT3 signaling pathways [11]. Costunolide, an active sesquiterpene lactone, is derived from several medicinal plants, such as Saussurea lappa Decne and Laurus nobilis L. Jin et al. showed that costunolide inhibited osteosarcoma growth and metastasis by suppressing STAT3 activity [12]. Cryptotanshinone, an active quinoid diterpene, can be isolated from Salvia miltiorrhiza Bunge, which is a famous Chinese herb also known as Danshen. Vundavilli et al. showed antitumor effects of cryptotanshinone against human osteosarcoma cell lines [13].

Oridonin is a kaurene-type diterpenoid that can be isolated from the Chinese herb Rabdosia rubescens. Kazantseva et al. showed that oridonin could function as a sensitizer to upregulate the antitumor effects of doxorubicin in human osteosarcoma cell lines by apoptosis and mitochondria dysfunction [14]. Butein is a chalcone derivative that can be isolated from various plants, including Rhus verniciflua Stokes, Semecarpus anacardium, and Dalbergia odorifera. Hsu et al. showed that butein could suppress cell proliferation and trigger senescence through ROS-dependent p53 activation in osteosarcoma U-2 OS cells [15]. Hinokitiol is a natural monoterpenoid that can be isolated from the heartwood of several Cupressaceae trees, such as Chamaecyparis obtusa, Thuja plicata, and Thujopsis dolabrata var. hondai. Yang et al. showed that hinokitiol exposure resulted in cell cycle arrest at the S phase and DNA damage response in both the U-2 OS and MG-63 osteosarcoma cell lines. Furthermore, in U-2 OS cells with wild-type tumor suppressor p53, hinokitiol exposure triggers a p53-dependent cellular senescence. However, in MG-63 cells with mutated p53, hinokitiol exposure significantly promotes cell apoptosis, not senescence. Suppression of autophagy by chloroquine enhances cellular senescence in U-2 OS cells and increases apoptosis in MG-63 cells [16].

Carvacrol is a monoterpenoid phenol that is abundantly present in the essential oils of many medicinal plants, including oregano (O. vulgare), thyme (T. vulgris), pepperwort (Lepidium flavum), and wild bergamot (Citrus aurantium var. bergamia Loisel) [17]. It is used as a chemical flavoring in various foods, such as alcoholic beverages. Several studies have indicated that carvacrol has excellent activities to inhibit the growth of microbes and viruses [17,18]. In addition, it has been demonstrated that carvacrol has anti-inflammatory activities and benefits wound healing by modulating inflammatory cytokines and improving re-epithelialization, angiogenesis, and collagen deposition [19,20]. Recent literature has shown its anticancer effects in several cancer cells. Carvacrol inhibits cell proliferation and induces apoptosis in cervical cancer via mediating MEK/ERK activation [21]. Carvacrol suppresses the expressions of matrix metalloproteinase-2/9 (MMP2/9) and triggers cell cycle arrest in G2/M phase and apoptosis to inhibit colon cancer proliferation [22]. Carvacrol inhibits the proliferation of human breast cancer MCF-7 cells via p53-dependnet apoptosis [23].

Reactive oxygen species (ROS) are highly reactive molecules that have essential roles in cellular signal transduction and homoeostasis. They can modulate various biological processes, such as cell proliferation, differentiation, and angiogenesis. However, excessive levels of ROS can cause cellular damage, leading to activation of the cell death machinery [24]. ROS-dependent cell death has been shown to be involved in the antitumor effect of different chemotherapeutic agents [25]. For example, cisplatin induces apoptosis via ROS overgeneration in osteosarcoma MG-63 cell line [26], methotrexate triggers cell death by upregulating ROS production in osteosarcoma U-2 OS cells [27], and carvacrol triggers ROS-dependent apoptosis to suppress proliferation in prostate cancer [28] and lung adenocarcinoma cell lines [29].

Endoplasmic reticulum (ER) is a membrane system of cells, and it is responsible for intracellular calcium balance, protein biosynthesis, protein folding, and transporting of synthesized proteins. When misfolded and unfolding proteins accumulate in the ER lumen, ER stress occurs and unfolded protein response (UPR) is activated to modulate proper protein folding and degradation of unfolded proteins [30]. Three ER stress protein sensors, namely, IRE1, PERK, and ATF6, are involved in UPR initiation. The three ER stress sensors are normally bound by the ER resident chaperone GRP78, which maintains them in an inactive state. Under ER stress, the three ER stress sensors are released from GRP78 and thus activate downstream signaling cascades to restore ER homeostasis [31]. However, if it fails to cope with unfolded proteins, UPR triggers cell death [32]. 4-Phenylbutyric acid (4-PBA) is an FDA-approved drug for urea cycle disorders. It can also act as an inhibitor of ER stress by promoting protein folding [33].

Several studies have shown that carvacrol has excellent activities to modulate different biological processes. However, few studies have explored its antitumor effect on osteosarcoma. In this report, we tried to estimate the potential efficacy of carvacrol against osteosarcoma cell lines and reveal the roles of ROS and ER stress in this effect.

## 2. Materials and Methods

### 2.1. Cell Culture and Chemical

Osteosarcoma HOS and U-2 OS cells were obtained from the Bioresource Collection and Research Center (Hsinchu, Taiwan). HOS and U-2 OS cell lines were cultured in MEM and McCoy’s 5A medium, respectively, with 10% fetal bovine serum and 100 U/mL penicillin/streptomycin at 37 °C in a 5% CO_2_ incubator. Carvacrol (purity ≥98%) and sodium 4-phenylbutyrate (4-PBA, purity ≥98%) were obtained from Cayman Chemical Company (Ann Arbor, MI, USA).

### 2.2. Cell Viability Assay

To determine cell viability, HOS and U-2 OS cells were seeded at a density of 2 × 10^4^ and 2.5 × 10^4^/well, respectively, in 24-well dishes overnight. The cells were exposed to different concentrations of carvacrol for 24 or 48 h. Finally, 10 µL of MTT solution (5 mg/mL) was added to the wells, and the cells were then incubated at 37 °C for another 2 h. After that, the medium with MTT was withdrawn, and 200 μL of DMSO was used to dissolve formazan crystals. The absorbance of samples was then measured at 570 nm by a microplate reader (Molecular Devices, San Jose, CA, USA). The IC_50_ values were calculated using the “Forecast” function in Microsoft Excel.

### 2.3. Colony Formation Assay

The cells (1.5 × 10^5^/dish) were incubated with different concentrations of carvacrol (0, 0.5, 0.6, and 0.7 mM) in the absence or presence of 4-PBA (1, 3, and 5 mM) for 16 h. After that, 500 of the exposed cells were seeded into 35 mm dishes with fresh culture medium without carvacrol. After 10 days of culture, crystal violet was used to stain colonies before counting.

### 2.4. Cell Cycle Analysis

Cells were exposed to carvacrol (0, 0.5, 0.6, and 0.7 mM) for 24 h. The exposed cells were collected, fixed with 100% ethanol, and stored at −20 °C overnight. After that, cells were washed twice with PBS and then incubated with a solution containing 40 μg/mL PI and 50 μg/mL RNase I in PBS for 30 min at room temperature (RT) in the dark. Finally, DNA content was examined by flow cytometry (Beckman Coulter Inc., San Jose, CA, USA).

### 2.5. Western Blotting

The cells were suspended in cold lysis buffer (10 mM Tris, pH 7.5, 1 mM EDTA, 420 mM NaCl, 10% glycerol, 0.5% NP-40 containing proteases inhibitor, and 1 mM DTT) and incubated on ice for 15 min. After that, the cell lysates were hardly centrifuged for 10 min at 4 °C, and the supernatants were collected and boiled in protein sample buffer (0.7 M β-mercaptoethanol, 4% SDS, 0.17 M Tris, pH 6.8, 0.17 mg/mL bromophenol blue, and 10% glycerol). The boiled samples were analyzed by SDS polyacrylamide gels. The resolved proteins were transferred onto the nitrocellulose membrane. The membranes were blocked in 1% milk in PBST buffer (PBS with 0.05% Tween 20) for 30 min and incubated with primary antibody for 2 h at RT. The membranes were washed three times by PBST buffer with 0.5% milk and then incubated with appropriate HRP-linked secondary antibody for 1 h at RT. After washing, the bound antibodies were detected by chemiluminescence reagents (Millipore, Burlington, MA, USA). The primary antibodies used are as follows: PARP (#9542; Cell signaling, Danvers, MA, USA), caspase 3 (#9662; Cell signaling), GRP78 (#3177; Cell signaling), IRE1a (#3294; Cell signaling), PERK (#5683; Cell signaling), CHOP (#2895; Cell signaling), GAPDH (#2118; Cell signaling), and beta-actin (NB600-501, Novus Biologicals, Littleton, CO, USA).

### 2.6. Apoptosis Cell Assay

Apoptotic cells were determined by the annexin V/PI double staining kit (Becton Dickinson, San Jose, CA, USA). Initially, HOS and U-2 OS cells were seeded at a density of 1 × 10^5^ and 1.3 × 10^5^/35 mm dish, respectively. The cells were incubated with carvacrol (0, 0.5, 0.6, and 0.7 mM) for 24 or 48 h. After that, the cells were washed, harvested, and stained with annexin V-FITC and PI according to the manufacturer’s protocol and analyzed by flow cytometry (Beckman Coulter Inc., San Jose, CA, USA).

### 2.7. Measurement of Intracellular Reactive Oxygen Species (ROS)

The cell permeant reagent 2′,7′–dichlorofluorescin diacetate (DCFDA) (Abcam, Cambridge, UK) was used to quantify the levels of ROS in live cell samples. Cells were exposed to 0.5 mM of carvacrol in the presence or absence of N-acetyl-L-cysteine for 24 h. These exposed cells were trypsinized, washed once by PBS, suspended, and incubated with 500 μL of PBS containing DCFDA (0.5 µM) for 20 min at 37 °C. Finally, the intracellular fluorescence was measured by flow cytometry (Beckman Coulter Inc., San Jose, CA, USA).

### 2.8. Statistical Analysis

All results are presented as mean ± standard error of mean (SEM) and were analyzed using Dunnett’s multiple comparisons test and Sidak’s multiple comparisons test for significant differences. *p* value < 0.05 was considered statistically significantly.

## 3. Results

### 3.1. Carvacrol Treatment Suppresses Cell Viability of Osteosarcoma Cell Lines

**Figure 1:**
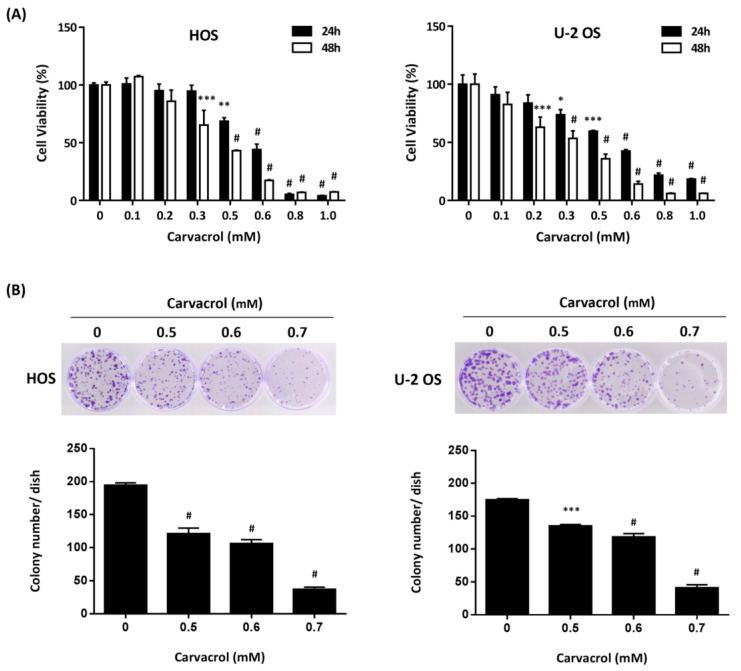
Carvacrol represses the growth of osteosarcoma cell lines. (**A**) Carvacrol represses cell growth of HOS and U-2 OS cells. HOS and U-2 OS cells were exposed to different concentrations of carvacrol for 24 or 48 h, and cell viability was estimated by MTT assay. Data are presented as mean ± S.E.M. of three independent experiments. (**B**) Carvacrol represses colony formation of HOS and U-2 OS cells. The cells were exposed to carvacrol (0.5, 0.6, and 0.7 mM) for 16 h. After that, 500 cells of the exposed cells were seeded into 35 mm dishes with fresh culture medium without carvacrol. After 10 days of culture, crystal violet was used to stain colonies, and the number of colonies was counted. Results are presented as mean ± S.E.M. (*n* = 3). * *p* < 0.05, ** *p* < 0.01, *** *p* < 0.001, # *p* < 0.0001 compared to the 0 mM group.

We first examined the effects of carvacrol on viability and proliferation of human osteosarcoma cell lines using the MTT assay. As shown in Figure 1A, carvacrol exposure apparently reduced cell viabilities of osteosarcoma HOS and U-2 OS cells (IC_50_ = 0.56 mM for HOS cells and 0.56 mM for U-2 OS cells at 24 h; IC_50_ = 0.47 mM for HOS cells and 0.39 mM for U-2 OS cells at 48 h). In addition, colony formation assay was used to examine the effect of carvacrol on single-cell proliferative capability of osteosarcoma cell lines. The results showed that carvacrol exposure decreased the colony number at the concentrations of 0.5, 0.6, and 0.7 mM in HOS and U-2 OS cells (Figure 1B). These results demonstrate that carvacrol could have antiproliferative effects against osteosarcoma cell lines.

**Figure 2:**
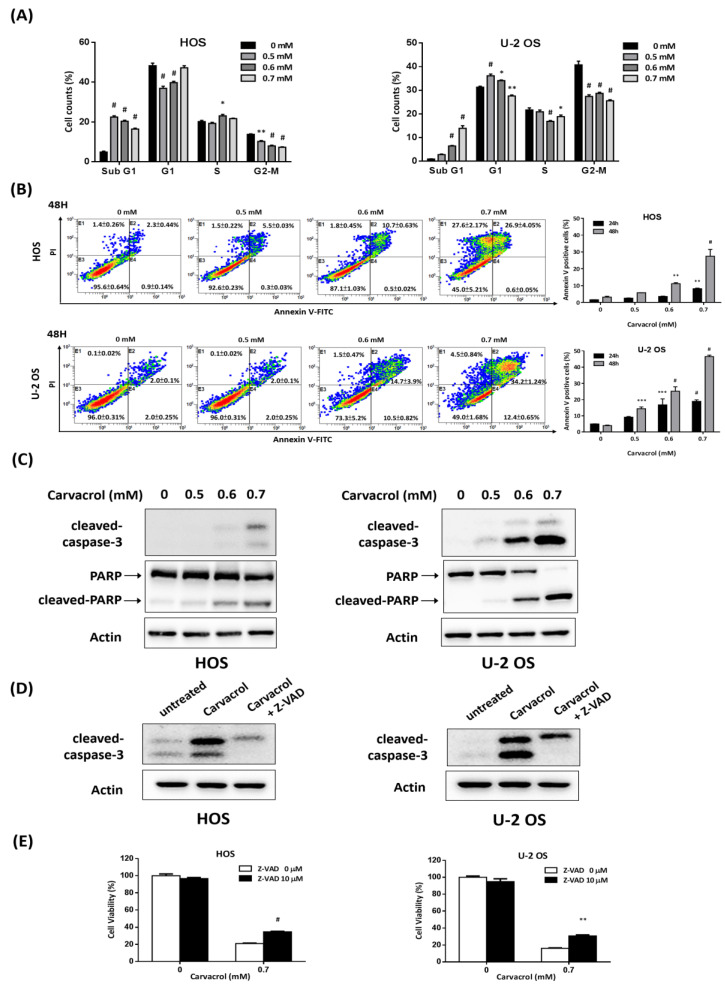
Carvacrol induces cell apoptosis in osteosarcoma cell lines. (**A**) Carvacrol exposure leads to accumulation of sub-G1 population in HOS and U-2 OS cells. The cells were exposed to different doses of carvacrol (0.5, 0.6, and 0.7 mM) for 24 h. To measure DNA contents, the cells were stained with propidium iodide (PI) and subsequently analyzed by flow cytometry. Data are presented as mean ± S.E.M. of three independent experiments. (**B**) Carvacrol exposure induces annexin V-positive cells in HOS and U-2 OS cells. The cells were exposed to different doses of carvacrol for 24 or 48 h. To analyze apoptotic cells, the cells were stained with annexin V and PI and analyzed using flow cytometry. Representative dot plots (48 h) and quantitative analysis of apoptotic cells (annexin V-positive cells) are shown. Data are presented as mean ± S.E.M. of three independent experiments. (**C**) Carvacrol exposure increases the levels of cleaved caspase 3 and cleaved PARP in osteosarcoma cell lines. The cells were exposed to carvacrol for 24 h. After that, the cells were analyzed by Western blotting using indicated antibodies by independent duplicate experiments. * *p* < 0.05, ** *p* < 0.01, *** *p* < 0.001, # *p* < 0.0001 compared to the 0 mM group. (**D**) Caspase inhibitor decreases the levels of cleaved caspase 3 in carvacrol-treated osteosarcoma cell lines. HOS or U-2 OS cells were exposed to carvacrol (0.7 mM) for 24 h in the presence or absence of Z-VAD-FMK (10 µM). After that, the exposed cells were analyzed by Western blotting using indicated antibodies by independent duplicate experiments. (**E**) Caspase inhibitor partly rescues the antiproliferative effect of carvacrol treatment. HOS and U-2 OS cells were exposed to carvacrol (0.7 mM) for 48 h, and cell viability was estimated by MTT assay. Data are presented as mean ± S.E.M. of three independent experiments. ** *p* < 0.01, # *p* < 0.0001 as compared with the Z-VAD 0 µM group.

To examine whether apoptosis is involved in the antigrowth effect of carvacrol, populations of sub-G1 on DNA content, the marker of apoptotic cells, were estimated by flow cytometry in carvacrol-treated osteosarcoma cell lines. As shown in Figure 2A, populations of sub-G1 were significantly upregulated after carvacrol treatment (0.5–0.7 mM) for 24 h in HOS and U-2 OS cells. Next, the apoptotic effects of carvacrol were verified by annexin V/PI double staining using flow cytometry. As shown in Figure 2B, the percentage of annexin-V positive cells in carvacrol-exposed cells were significantly upregulated. Furthermore, the levels of cleaved caspase 3 and PARP, the apoptosis-related proteins, were examined by Western blotting. As shown in Figure 2C, carvacrol exposure for 24 h increased the levels of cleaved caspase 3 and PARP in a dose-dependent manner. Moreover, the caspase inhibitor Z-VAD-FMK decreased the levels of cleaved caspase 3 in carvacrol-treated osteosarcoma cell lines (Figure 2D) and partly rescued the antiproliferative effect of carvacrol treatment in these cell lines (Figure 2E). The data indicate that carvacrol exposure can trigger apoptosis in osteosarcoma cell lines. In addition, our data showed that high dose of carvacrol (0.7 mM) resulted in an increase in PI-positive but annexin V-negative cells in HOS cells (Figure 2B), suggesting that the dose of carvacrol could induce nonapoptotic cell death in this cell line.

### 3.2. ROS Is Involved in Carvacrol-Induced Apoptosis in Osteosarcoma Cell Lines

**Figure 3:**
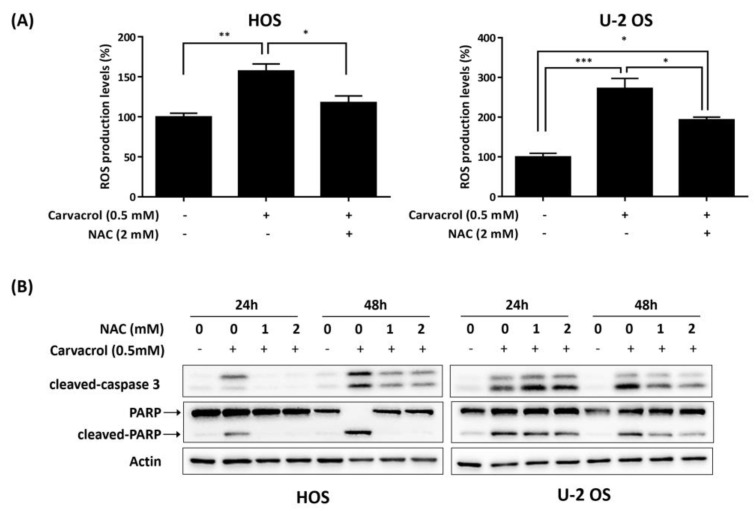
ROS is involved in carvacrol-induced apoptosis in osteosarcoma cell lines. (**A**) Carvacrol treatment increases the levels of ROS in HOS and U-2 OS cells. The cells were exposed to carvacrol in the presence or absence of N-acetyl-L-cysteine (NAC) for 24 h, and ROS levels were then measured by flow cytometry using 2′,7′-dichlorofluorescin diacetate (DCFDA) staining. Data are presented as mean ± S.E.M. of three independent experiments. * *p* < 0.05, ** *p* < 0.01, *** *p* < 0.001 when compared between groups. (**B**) Attenuation of ROS levels by NAC suppresses the levels of cleaved caspase 3 and cleaved PARP in carvacrol-exposed osteosarcoma cell lines. The cells were exposed to carvacrol in the presence or absence of NAC for 24 or 48 h. After that, the cells were analyzed by Western blotting using indicated antibodies by independent duplicate experiments.

Evidence indicates that reactive oxygen species (ROS) could regulate cell apoptosis [34]. We estimated the cellular redox status after carvacrol exposure for 24 h in osteosarcoma cell lines using DCFDA, a fluorogenic dye that assesses ROS within the cell. As shown in Figure 3A, carvacrol exposure significantly upregulated the levels of ROS in HOS and U-2 OS cells. Furthermore, coadministration of N-acetyl-L-cysteine (NAC), a ROS scavenger, resulted in attenuation of the increased cleaved caspase 3 and PARP in these cells (Figure 3B). The results suggest that ROS may be involved in carvacrol-induced apoptosis in osteosarcoma HOS and U-2 OS cells.

### 3.3. Attenuation of ER Stress Enhances the Antitumor Activity of Carvacrol in Osteosarcoma Cell Lines

**Figure 4:**
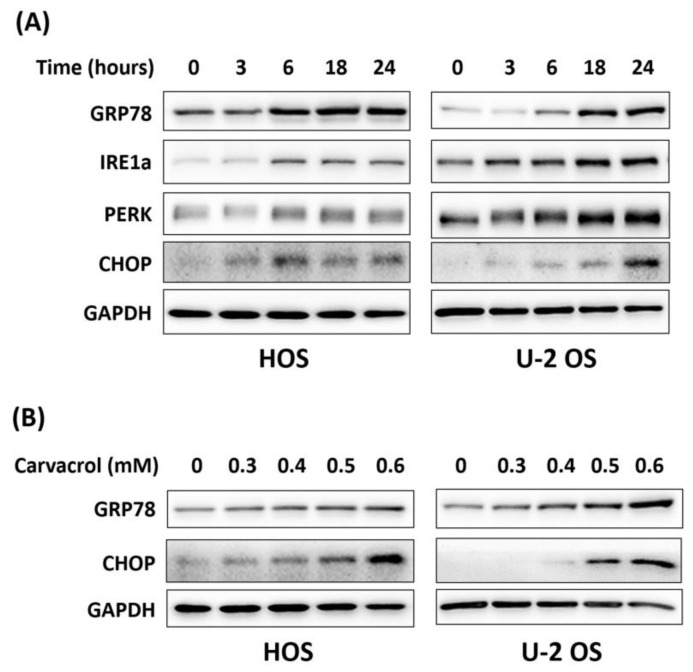
Carvacrol exposure induces ER stress in osteosarcoma cell lines. The cells were exposed to 0.5 mM of carvacrol and collected in the indicated timepoint (**A**) or incubated with different doses of carvacrol for 24 h (**B**). Expression of ER stress markers were analyzed in exposed cells by Western blotting. Data were obtained by independent duplicate experiments.

**Figure 5:**
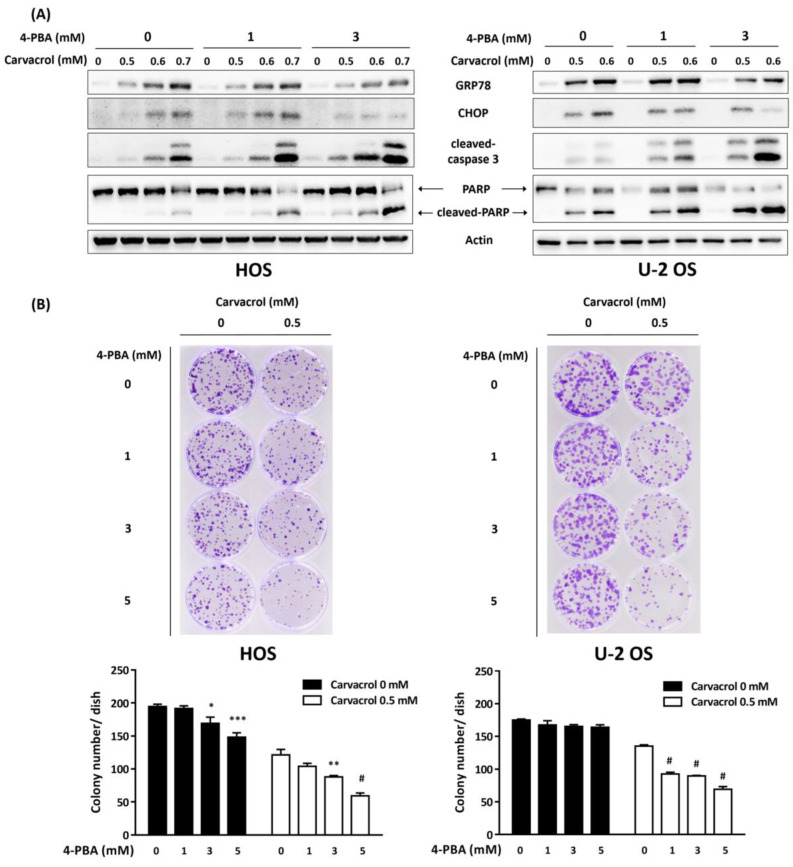
Attenuation of ER stress enhances the antitumor activity of carvacrol against osteosarcoma cell lines. (**A**) Coadministration of ER stress inhibitor 4-PBA enhances carvacrol-induced apoptosis. HOS or U-OS cells were exposed to carvacrol for 24 h in the presence or absence of 4-PBA. After that, the exposed cells were analyzed by Western blotting using indicated antibodies by independent duplicate experiments. (**B**) Attenuation of ER stress suppresses the colony formation in carvacrol-exposed cells. The cells were exposed to carvacrol for 16 h in the presence or absence of 4-PBA. After that, the exposed cells were plated in colony formation assay, and the number of colonies was counted. Results are presented as mean ± S.E.M. (*n* = 3). * *p* < 0.05, ** *p* < 0.01, *** *p* < 0.001, # *p* < 0.0001 compared to the 0 mM group.

In this report, we also observed that carvacrol exposure resulted in increased expression of ER stress markers, including GRP78, IRE1a, PERK, and CHOP, in osteosarcoma HOS and U-2 OS cells (Figure 4). This indicates carvacrol could have the ability to trigger ER stress in osteosarcoma cell lines. However, ER stress has been found to both protect cells from apoptosis and to activate apoptosis in these cells [35]. To determine the roles of ER stress in carvacrol-induced apoptosis, 4-PBA, an ER stress inhibitor, was coadministrated in these cells. As shown in Figure 5A, coadministration of 4-PBA at a higher dose (3 mM) resulted in attenuation of ER stress markers and simultaneously increased the levels of cleaved caspase-3 and cleaved PARP. In addition, coadministration of 4-PBA further suppressed clonogenic activity in carvacrol-exposed osteosarcoma cell lines (Figure 5B). The results indicate that ER stress triggered by carvacrol exposure protect cells from apoptosis and its attenuation enhances the antitumor activity of carvacrol in osteosarcoma cell lines.

## 4. Discussion

Various anticancer compounds show the ability to trigger endoplasmic reticulum (ER) stress. The stimulated ER stress either enhances cancer cell death or protects cells from apoptosis, which acts as a mechanism of resistance to chemotherapy. For instance, α-mangostin, a natural compound isolated from mangosteen, induces apoptosis and ER stress in osteosarcoma cell lines. Cotreatment with 4-PBA (an ER stress inhibitor) suppresses α-mangostin-induced apoptosis in these cells, suggesting that α-mangostin-induced ER stress positively contributes to apoptosis in these cells [36]. On the contrary, coadministration of 4-PBA reduces the expression of GRP78 and CHOP and enhances apoptosis in cisplatin-treated osteosarcoma cell lines, indicating that cisplatin-induced ER stress protects cell from apoptosis in these cell lines [37]. Khan et al. showed that carvacrol nanoemulsion could induce apoptosis and ER stress in lung adenocarcinoma A549 cells [29]. However, the link between apoptosis and ER stress in carvacrol-treated lung cancer cells is unclear. In this report, our data demonstrated that carvacrol could have the potential for triggering ER stress in osteosarcoma cell lines and that coadministration of the ER stress inhibitor 4-PBA enhanced carvacrol-induced apoptosis in osteosarcoma cell lines, indicating that upregulated ER stress protects osteosarcoma cells from carvacrol cytotoxicity.

Reactive oxygen species (ROS) generation has been linked to ER stress. ROS can be produced in ER as byproducts of protein folding, and ER stress in some conditions upregulates the levels of ROS in the ER or mitochondria. In addition, overwhelming ROS levels have been shown to influence ER protein folding and induce ER stress [38]. Therefore, it is suggested that ROS generation could be downstream or upstream of ER stress. In this report, we demonstrated that carvacrol exposure induced ER stress and increased ROS levels in osteosarcoma cell lines, but the interaction between ER stress and ROS needs to be further investigated in these cells.

The literature shows that various concentrations of carvacrol could induce cytotoxic effect in different cancer cells. It was found that 0.5 to 1 mM of carvacrol significantly suppressed cell growth in the nonsmall lung cancer cell line A549 [39], while 0.3 to 0.7 mM of carvacrol induced cell cycle arrest and apoptosis in the colon cancer cell lines HCT116 and LoVo [22]. In addition, higher than 0.3 mM carvacrol significantly induced apoptosis in several breast cancer cell lines, including BT-474, MDA-MB-231, and MCF-7 cells [40]. In this report, the data showed that 0.3 to 1 mM of carvacrol was effective concentration to inhibit proliferation of osteosarcoma HOS and U-2 OS cell lines. Recently, some potential ways to enhance antitumor activity of carvacrol have been revealed, such as nanoemulsions for drug delivery [29] and coexposure to 5-FU [41] or X-radiation [42]. Based on our results, ER stress may be a target to enhance antitumor activity of carvacrol in osteosarcoma cell lines.

Several studies have successfully demonstrated the antitumor activities of carvacrol in in vivo experiments. For example, Sivaranjani et al. showed that administration of carvacrol significantly suppressed the tumor incidence and the number of colonic aberrant crypt foci in DMH (1,2-dimethylhydrazine)-induced colon carcinogenesis in male rats [43]. Subramaniyan et al. further demonstrated that carvacrol exposure suppressed the elevation of tumor marker levels and reduced mast cell density and cell proliferation in a DEN (diethylnitrosamine)-induced hepatocarcinogenesis rat model [44]. In addition, a carvacrol nanoemulsion was found to demonstrate anticancer activity against human lung adenocarcinoma A549 cell line in an in vivo xenograf mice model [29]. In this report, our data revealed that carvacrol significantly suppressed cell viabilities of osteosarcoma HOS and U-2 OS cell lines, and the inhibition of ER stress by 4-PBA enhanced its anticancer activity in in vitro studies. Whether these effects could be verified in in vivo assays should be further addressed in the future. Furthermore, carvacrol should be compared to other standard drugs, such as doxorubicin or cisplatin, at the same time to understand the efficacy of drug treatment.

Several studies have demonstrated that poor chemotherapy response is correlated with poor prognosis in osteosarcoma patients [4,45]. In addition, tumor relapse is a difficult issue for treating osteosarcoma. Once relapse occurs, the five-year post-relapse overall survival rate is very low at approximately 20 to 30% [7,8]. Moreover, in several reported studies, chemotherapy used for treating the first relapse was not associated with survival after recurrence [7,9,10]. Therefore, it is urgently necessary to develop effective drugs for patients suffering from osteosarcoma. Some anticancer drugs have been evaluated for treating osteosarcoma in clinical trials, such as pemetrexed and carboplatin [46]. In this report, we demonstrated the antitumor activity of carvacrol against osteosarcoma and revealed that the inhibition of carvacrol-induced ER stress by 4-PBA enhanced the antitumor activity of carvacrol. We hope our findings can contribute to the further development of carvacrol as anticancer drugs for treating osteosarcoma.

In conclusion, this study showed that carvacrol exposure significantly inhibited cell viabilities, increased ROS generation, and resulted in apoptosis in osteosarcoma cell lines. In addition, we found that carvacrol-induced ER stress protected cells from apoptosis and that its attenuation enhanced the antitumor activity of carvacrol in osteosarcoma cell lines. These results provide an insight into the mechanism of carvacrol action and suggest potential therapeutic strategies for osteosarcoma.

## Data Availability

Data sharing not applicable—no new data generated.
